# Association of *MIF* rs1007888 and *ARAP1* rs1552224 genetic variants with the risk of gestational diabetes mellitus in a chinese population; case study and meta-analysis

**DOI:** 10.3389/fendo.2025.1650782

**Published:** 2025-09-16

**Authors:** Yuxuan Zhang, Yanying Wu, Qiaoli Zeng, Watson Ray Gyan, Sining Huang, Xiner Dai, Jia Liu, Xin Liu, Yue Wei, Runmin Guo

**Affiliations:** ^1^ Department of Internal Medicine, Shunde Women and Children’s Hospital (Maternity and Child Healthcare Hospital of Shunde Foshan), Guangdong Medical University, Foshan, Guangdong, China; ^2^ Maternal and Child Research Institute, Shunde Women and Children’s Hospital (Maternity and Child Healthcare Hospital of Shunde Foshan), Guangdong Medical University, Foshan, Guangdong, China; ^3^ Key Laboratory of Research in Maternal and Child Medicine and Birth Defects, Guangdong Medical University, Foshan, Guangdong, China; ^4^ Department of Ultrasound, Shunde Women and Children’s Hospital, Maternity and Child Healthcare Hospital of Shunde Foshan, Guangdong Medical University, Foshan, Guangdong, China

**Keywords:** macrophage migration inhibitory factor (MIF), Ankyrin Repeat and PH Domain1 (*ARAP1*), Rs1007888, rs1552224, gestational diabetes mellitus

## Abstract

**Background:**

Macrophage migration inhibitory factor (*MIF*) rs1007888 is significantly associated with pancreatic *β*-cell function and insulin resistance in patients with gestational diabetes mellitus (GDM). The ArfGAP with RhoGAP domain, ankyrin repeat, and PH domain-containing protein 1 (*ARAP1*) rs1552224 locus has been identified as a risk locus for type 2 diabetes, and recent reports have linked it to elevated blood glucose levels and reduced insulin release upon glucose stimulation. Few studies have been conducted on these genetic variants and their risk of GDM. This study aimed to investigate the association between these two genetic variants (*ARAP1*) rs1552224 and (*MIF*) rs1007888 and the risk of developing GDM.

**Methods:**

A case-control study involving 500 GDM patients and 502 healthy controls was conducted. DNA was extracted, and rs1007888 and rs1552224 were systematically genotyped using the SNPscan™ genotyping kit. Statistical methods assessed genotype and allele differences linked to GDM risk, followed by a meta-analysis to evaluate the impact of regional factors on GDM.

**Results:**

Analyses of (*MIF*) rs1007888 showed no link to higher GDM risk, but meta-analysis found a significant association (OR>1), indicating a connection to increased GDM risk. *ARAP1* rs1552224 was significantly linked to reduced GDM incidence (Allele Model A vs. C: OR = 0.624; 95% CI: 0.425-0.916; *p*-value = 0.016; Dominant Model AA vs. AC+CC: OR = 0.641; 95% CI: 0.429-0.959; *p*-value = 0.030), especially in women under 30, rs1552224 Aelle Model (A vs. C: OR = 0.490; 95% CI: 0.281-0.857; *p* -value = 0.012), Dominant Model (AA vs. AC + CC: OR = 0.523; 95% CI: 0.292-0.938; *p* -value = 0.030). and those with a BMI≥24, Aelle Model (A vs. C: OR = 0.345; 95% CI: 0.124-0.960; *p*-value = 0.042). Conversely, a meta-analysis suggested an increased GDM risk with the *ARAP1* variant (OR>1).

**Conclusion:**

The meta-analysis results demonstrate that there is an enhanced likelihood of GDM associated with the *MIF* rs1007888 mutation. Moreover, our findings indicate that the *ARAP1* rs1552224 variant, specifically the AC genotype and C allele, confers a decreased risk of developing gestational diabetes mellitus (GDM). The outcomes obtained give GDM testing a theoretical foundation.

## 1 Introduction

Gestational diabetes mellitus (GDM) is a pregnancy-specific disorder of glucose metabolism, distinct from pre-existing diabetes, and is increasingly prevalent in China. It poses significant risks to maternal health, including preeclampsia, higher rates of cesarean delivery, and a markedly increased likelihood of developing type 2 diabetes mellitus (T2DM) later in life. Offspring of affected mothers are also at risk of adverse outcomes such as macrosomia, neonatal hypoglycemia, and long-term metabolic complications (1–3). Established risk factors for GDM include advanced maternal age, obesity, family history of diabetes, and genetic susceptibility (4).

Although the pathophysiology of GDM is not fully understood, it shares essential features with T2DM, including insulin resistance, impaired glucose tolerance, and β-cell dysfunction (2, 3). Increasing evidence points to the contribution of genetic variation, particularly single-nucleotide polymorphisms (SNPs), in modulating GDM risk. Among candidate loci, the macrophage migration inhibitory factor (*MIF*) gene and the *ARAP1* locus have been implicated in diabetes-related traits. *MIF* rs1007888 has been associated with insulin resistance and β-cell dysfunction (5, 6), while *ARAP1* rs1552224 has been linked to reduced insulin secretion (7). However, their roles in GDM remain insufficiently investigated.

*MIF* is a pleiotropic cytokine expressed abundantly by placental trophoblasts and upregulated in GDM placental tissue, correlating with fasting glucose levels and insulin resistance (8, 9). Functionally, *MIF* influences insulin secretion and glucose metabolism and exerts pro-inflammatory effects by regulating cytokines such as TNF-α, IFN-γ, and IL-10, thereby contributing to hyperglycemia (9). Elevated systemic *MIF* levels are a feature of T2DM (10), but its mechanistic role in pregnancy-related glucose dysregulation remains poorly defined (8).

Meanwhile, genome-wide association studies (GWAS) consistently implicate *ARAP1* variants in T2DM susceptibility (11, 12). In particular, rs1552224 has been associated with fasting glucose levels and impaired glucose-stimulated insulin secretion, with evidence suggesting the A allele contributes to β-cell dysfunction (11). Interestingly, preliminary data indicate that this allele may be protective against GDM (13), highlighting potential differences between pregnancy-related and non-pregnancy-related diabetes risk mechanisms.

Despite these insights, data on the association between *MIF* rs1007888 and *ARAP1* rs1552224 with GDM remain sparse, especially in Asian populations, and no systematic synthesis of existing findings has been undertaken. Given the high burden of GDM and the distinct genetic architecture of the Chinese population, further investigation is warranted.

Therefore, in this study, we examined the associations of *MIF* rs1007888 and *ARAP1* rs1552224 with GDM in a Han Chinese cohort and performed a meta-analysis to contextualize our findings with the broader literature. We aimed to clarify the potential contribution of these variants to GDM risk and provide new insights into their role in pregnancy-specific glucose metabolism.

## 2 Materials and methods

### 2.1 Study participants

A total of 1002 participants were recruited for the study, including 500 patients with gestational diabetes mellitus (GDM) and 502 pregnant women without GDM who served as controls. This study protocol was approved by the Ethics Committee of Shunde Women’s and Children’s Hospital Affiliated to Guangdong Medical University (approval ID: 2020072). The inclusion criteria were as follows: participants must provide voluntarily signed informed consent; be of Han ethnicity; be at least 18 years old; have undergone a 75g oral glucose tolerance test (OGTT) between 24 and 28 weeks of gestation and be diagnosed with gestational diabetes mellitus (GDM) or have standard glucose tolerance according to the criteria of the International Association of Diabetes and Pregnancy Study Groups (IADPSG). In the present study, the International Association of Diabetes and Pregnancy Investigation Groups (IADPSG) diagnostic guidelines were employed. If one or more points satisfy the following criteria, GDM was diagnosed: fasting blood glucose (FBG) ≥ 5.1 mmol/L, 1-hour postprandial glucose (PG) ≥ 10.0 mmol/L, or 2-hour PG ≥ 8.5 mmol/L. Expectant mothers falling below these specified limits were classified as healthy control subjects.

The exclusion criteria included: presence of pregnancy-related diseases or use of drugs affecting glucose metabolism; history of severe cardiovascular and cerebrovascular diseases, hepatic or renal insufficiency, tumors, or pathogenic infections; and engagement in smoking, alcoholism, drug abuse, or presence of intellectual disability or mental disorders. This study was conducted following the principles outlined in the Declaration of Helsinki.

### 2.2 Data collection

During the 24–28 gestational weeks, comprehensive data were collected, including parity (primigravida or multigravida), pre-pregnancy weight, ethnicity, age, height, blood pressure, and blood glucose levels. The collected data were subsequently employed to compute the pregestational body mass index (pre-BMI, kg/m²), which was defined as the pregestational weight (in kilograms) divided by the square of height (in meters). To determine the obesity status in line with Chinese standards, the following classification criteria were utilized: underweight (< 18.5 kg/m²), normal weight (18.5–24.9 kg/m²), overweight (25–29.9 kg/m²), and obese (≥ 29 kg/m²). This method of classification was adopted based on the research carried out by (14).

### 2.3 SNP genotyping and quality control

Based on findings from genome-wide association studies (GWAS) of type 2 diabetes mellitus (T2DM) in Asian populations, we selected two candidate SNPs, *MIF* rs1007888 and *ARAP1* rs1552224, for evaluation of their potential association with GDM. Selection criteria included a minor allele frequency (MAF) > 0.05, supported by evidence from prior studies (8, 15, 16).

For each participant, 2 mL of peripheral blood was collected into EDTA tubes and stored at −80 °C until analysis. Genomic DNA was extracted using the QIAamp DNA Blood Kit (Qiagen, Germany) according to the manufacturer’s protocol. SNP genotyping was performed using the SNPscan™ method (Genesky Biotechnologies, Shanghai, China), a high-throughput and highly accurate technique based on dual ligation probe hybridization and multiplex fluorescent PCR. The procedure involves probe ligation to discriminate wild-type and variant alleles, multiplex PCR amplification with fluorescently labeled primers, and capillary electrophoresis to separate amplified fragments. Genotypes were assigned based on fragment length and fluorescent signal intensity.

To ensure accuracy, rigorous quality control measures were implemented by Genesky Biotechnologies (12, 17). Pre-experiments were conducted to optimize assay performance. In addition, 6% of randomly selected samples were re-genotyped by Sanger sequencing, yielding 100% concordance with the SNPscan results.

### 2.4 Statistical analyses

The analytical evaluations were performed via SPSS 20.0 (SPSS Inc., Chicago, IL, USA), with a bilateral *p*-value less than 0.05 considered statistically significant. Standard distribution-aligned variables were recorded as means ± standard deviations, and the non-overlapping samples t-test was used to assess the variances in relevant parameters between the two groups. When the normality assumption was violated, non-parametric testing methods were employed. Descriptive data were evaluated using the chi-square (χ^2^) test. To verify the representativeness of the control group in the population, the Hardy-Weinberg equilibrium (HWE) test, estimated by the goodness-of-fit χ^2^, was applied. Six genetic models, specifically codominant homozygous, codominant heterozygous, over-dominant, dominant, recessive, and allele models, were used to assess GDM risk through the χ^2^ test and logistic regression analysis. The presentation included basic and adjusted odds ratios (ORs) along with their respective 95% confidence intervals (CIs), considering variables such as age, pre-BMI, blood pressure, parity, and more. A stratified analysis was performed to delve deeper into how age and pre-BMI might affect the outcomes. A one-way ANOVA was used to explore the link between SNPs and blood sugar levels. The least significant difference (LSD) approach was adopted for multiple comparisons. Investigations of specific subgroups were conducted for both GDM and T2DM.

### 2.5 Meta-analysis

A thorough literature review and meta-analysis were conducted utilizing the Google Scholar, PubMed, and CNKI databases to assess the association between the *MIF* rs1007888 and *ARAP1* rs1552224 polymorphisms and the risk of gestational diabetes mellitus (GDM) and type 2 diabetes mellitus (T2DM) (**Supplementary Table 7**). A limited number of studies were available for the meta-analysis concerning these genes and their associated polymorphisms. The authors propose further studies with these genes. For the analysis of rs1552224 concerning GDM, one eligible study was included, while three studies were incorporated for rs1552224 concerning T2DM. Additionally, four studies were selected for the association between *MIF* rs1007888 and GDM (refer to **Supplementary Table 7**). The analyses were performed using a fixed-effects model.

The search strategy employed combinations of the terms rs1007888, rs1552224, type 2 diabetes mellitus (T2DM), and gestational diabetes mellitus (GDM). Eligible studies were those that focused on case-control or cohort analyses exploring the association between the rs1007888 and rs1552224 polymorphisms and T2DM or GDM, provided they contained sufficient original data. Studies that did not meet the established diagnostic criteria or deviated from the Hardy-Weinberg equilibrium were excluded from the analysis. Data extraction was carried out independently by two authors, with any discrepancies resolved through consultation with a third party. The kappa coefficient was calculated to evaluate inter-researcher agreement, ensuring the objectivity and accuracy of the study selection process.

Meta-analyses were performed across six genetic models, employing fixed-effects or random-effects models depending on the level of heterogeneity observed. Publication bias was assessed using Egger’s and Begg’s tests. All analyses were conducted using STATA version 16.0. This study adheres to the PRISMA guidelines and the Cochrane Handbook. The protocol has been registered with PROSPERO, an internationally recognized platform for the registration of systematic reviews and meta-analyses, under the registration number CRD420251122128.

## 3 Results

### 3.1 Overview of the clinical characteristics of the subjects

The study’s foundational analysis of 500 GDM patients and 502 non-diabetic controls (**Table 1**) revealed several critical baseline differences. Notably, the GDM cohort exhibited significantly higher mean values for key clinical markers, including systolic blood pressure (SBP), diastolic blood pressure (DBP), age, pre-pregnancy body mass index (pre-BMI), fasting plasma glucose (FPG), 1-hour postprandial glucose (1h-PG), and 2-hour postprandial glucose (2h-PG) when compared to the control group (all *p* < 0.05). This suggests that beyond the defining glucose dysregulation, the GDM group presents with a broader metabolic and physiological profile distinct from non-diabetic individuals, even at baseline. Furthermore, a significant disparity in parity was evident between the GDM and control groups (*p* < 0.05), indicating that reproductive history may serve as an additional differentiating factor. This finding warrants further exploration into its potential implications for GDM pathogenesis or its role as a risk indicator. Stratified analyses reinforced the robustness of these observations. When stratifying the cohort using age 30 and pre-BMI cut-off points of 18.5 and 24, significant differences between the GDM and control groups persisted (all *p* < 0.05). This reason suggests that broad population characteristics do not merely drive the observed disparities but remain significant even within specific demographic and anthropometric subgroups, emphasizing the pervasive nature of these differences in the GDM cohort.

**Table 1:** Fundamental and categorized traits of the study subjects.

Variables	Cases (%) (N=500)	Controls (%) (N=502)	t/x^2^	*P*
Age, year (mean ± SD)	31 ± 4	29 ± 4	-8.56	<0.001
					pre-BMI, kg/m2	21.51 ± 3.10	20.53 ± 2.58	-5.42	<0.001
SBP, mmHg	117 ± 11	114 ± 10	-3.53	<0.001					
					DBP, mmHg	70 ± 8	68 ± 7.3	-3.23	0.001
FPG, mmol/L	4.82 ± 0.64	4.50 ± 0.31	-9.75	<0.001					
					1h-PG, mmol/L	10.17 ± 1.60	7.66 ± 1.27	-26.22	<0.001
2h-PG, mmol/L	8.91 ± 1.60	6.69 ± 0.99	-25.85	<0.001					
					Parity (n)			8.88	0.003
Primipara	210 (42)	258(51.4)							
					Multipara	290(58)	244(48.6)		
Age, year			49.2	<0.001					
< 30	26.60 ± 2.06	25.82 ± 2.70		
≥ 30	33.75 ± 2.84	33.01 ± 2.42		
pre-BMI, kg/m2			27.8	<0.001
< 18.5	17.53 ± 0.86	17.65 ± 1.47		
18.5 ≤ BMI < 24	20.96 ± 1.49	20.67 ± 1.43		
≥ 24	26.14 ± 2.82	25.82 ± 3.24		

SBP, systolic blood pressure; DBP, diastolic blood pressure; pre-BMI, pre-gestational body mass index; FPG, fasting plasma glucose; 1h-PG, 1-hour postprandial glucose; 2h-PG, 2-hour postprandial glucose.

### 3.2 The relationship between genetic polymorphisms and GDM risk

#### 3.2.1 Overall analysis results

The control group’s genetic data provides crucial insights into the Hardy-Weinberg Equilibrium (HWE) of two specific SNPs, rs1007888 and rs1552224. These SNPs are located at chromosomal positions 11:72722053 and 22:23898914, respectively. For rs1007888, the major and minor alleles are C and A, with a Minor Allele Frequency (MAF) of 0.486. For rs1552224, the major and minor alleles are T and C, and its MAF is 0.079 (**Table 2**). Critically, the HWE test yielded high *p*-values for both SNPs (0.997 for rs1007888 and 0.86 for rs1552224), strongly indicating that both loci are in Hardy-Weinberg equilibrium within the studied population. This finding is fundamental, as it confirms that the control group is representative of an unperturbed genetic population, making it a reliable baseline for further genetic association studies.

**Table 2:** Information on SNPs and Hardy-Weinberg equilibrium (HWE) test among the controls.

SNP	Min/Maj	Chr. position	MAF	HWE (*P*)
rs1552224	C/A	chr11:72722053	0.079	0.86
rs1007888	T/C	chr22:23898914	0.486	0.997

SNP, single nucleotide polymorphisms; Min, minor allele; Maj, major allele; HWE, Hardy–Weinberg equilibrium; MAF, frequency of minor allele.

### 3.3 Association between SNPs and the risk of GDM in all of the subjects

In our study, even after accounting for potential confounding factors such as pre-BMI, systolic and diastolic blood pressure, maternal age, and parity, a robust association between the rs1552224 genetic variant and a reduced risk of GDM remained evident. Specifically, analysis within the dominant model (AA genotype compared to AC + CC genotypes) yielded an odds ratio of 0.641 (95% CI: 0.429-0.959; *p* = 0.030), while the allele model (A vs. C allele) showed an odds ratio of 0.624 (95% CI: 0.425-0.916; *p* = 0.016). These findings, detailed in **Table 3**, strongly suggest a protective effect of the variant against GDM. Conversely, our analysis revealed no significant correlation between the rs1007888 locus and GDM risk (**Table 3**).

**Table 3:** The correlations between SNPs and the risk of GDM in all of the subjects.

Model	Cases (%) (n=500)	Controls (%) (n=502)	Crude OR (95% CI)	Crude *P*	Adjusted OR (95% CI)	Adjusted *P*
rs1552224						
Codominant model						
AA	450(90.0)	427(85.1)	1(ref)		1(ref)	
AC	50(10.0)	71(14.1)	0.668(0.455-0.982)	0.04	0.680(0.453-1.020)	0.062
CC	0(0.0)	4(0.80)				
Aelle model						
A	950(95.0)	925(92.1)	1(ref)		1(ref)	
C	50(5.0)	79(7.9)	0.616(0.428-0.888)	0.009	0.624(0.425-0.916)	0.016
Dominant Model						
AA	450(90.0)	427(85.1)	1(ref)		1(ref)	
AC+CC	50(10.0)	75(14.9)	0.633(0.432-0.926)	0.019	0.641(0.429-0.959)	0.03
Recessive Model						
AC+AA	500(100.0)	498(99.2)	1(ref)		1(ref)	
CC	0(0.0)	4(0.80)	NA	NA	NA	NA
Overdominant model						
AA+CC	450(90.0)	431(85.9)	1(ref)		1(ref)	
AC	50(10.0)	71(14.1)	0.674(0.459-0.991)	0.045	0.686(0.457-1.029)	0.069
rs1007888						
Codominant model						
CC	152(30.4)	133(26.5)	1(ref)		1(ref)	
CT	230(46.0)	250(49.8)	0.805(0.600-1.080)	0.148	0.783(0.573-1.071)	0.126
TT	118(23.6)	119(23.7)	0.868(0.615-1.225)	0.42	0.867(0.603-1.249)	0.444
Aelle model						
C	534(53.4)	516(51.4)	1(ref)		1(ref)	
T	466(46.6)	488(48.6)	0.923(0.774-1.100)	0.369	0.931(0.774-1.121)	0.452
Dominant Model						
CC	152(30.4)	133(26.5)	1(ref)		1(ref)	
CT+TT	348(69.6)	369(73.5)	0.825(0.627-1.086)	0.171	0.813(0.607-1.087)	0.162
Recessive Model						
CT+CC	382(76.4)	383(76.3)	1(ref)		1(ref)	
TT	118(23.6)	119(23.7)	0.994(0.743-1.331)	0.969	1.037(0.763-1.410)	0.816
Overdominant model						
CC+TT	270(54.0)	252(50.2)	1(ref)		1(ref)	
CT	230(46.0)	250(49.8)	0.859(0.670-1.100)	0.229	0.822(0.632-1.069)	0.144

By adjusting for pre-pregnancy body mass index, Systolic blood pressure, Diastolic blood pressure, age, and parity, logistic regression yielded the adjusted *p*-value.

### 3.4 Stratified analysis of the differences between SNPs in the *ARAP1* and *MIF* genes and GDM risk in subjects under 30 years of age.

Our stratified analysis across six genetic models, meticulously accounting for age and pre-BMI, revealed a compelling association between specific SNPs and GDM risk in subjects under 30 years of age. Notably, the *ARAP1* rs1552224 allele model demonstrated a significant protective effect, correlating with a substantially lower incidence of GDM in women under 30 years of age. This association remained robust even after comprehensive adjustment for potential confounding factors (A vs. C: OR = 0.490, 95% Confidence Interval: 0.281-0.857; *p* = 0.012). Further supporting this finding, the dominant model (AA vs. AC + CC: OR = 0.523, 95% Confidence Interval: 0.292-0.938; *p* = 0.030) also indicated a statistically significant reduction in GDM risk within this younger subgroup (**Table 4**). These results suggest that *ARAP1* rs1552224 warrants further investigation as a potential genetic marker for GDM susceptibility, particularly in younger individuals.

**Table 4:** The connections between SNPs in the *ARAP1* and *MIF* genes and GDM risk in subjects under 30 years of age.

Model	Cases (%) (n=192)	Controls (%) (n=304)	Crude OR (95% CI)	Crude *P*	Adjusted OR (95% CI)	Adjusted *P*
rs1552224						
Codominant model						
AA	173(90.1)	254(83.6)	1(ref)		1(ref)	
AC	19(9.9)	46(15.1)	0.606(0.344-1.071)	0.085	0.585(0.324-1.053)	0.074
CC	0()	4(1.3)	()		()	
Aelle model						
A	365(95.1)	554(91.1)	1(ref)		1(ref)	
C	19(4.9)	54(8.9)	0.534(0.311-0.916)	0.023	0.490(0.281-0.857)	0.012
Dominant Model						
AA	173(90.1)	254(83.6)	1(ref)		1(ref)	
AC+CC	19(9.9)	50(16.4)	0.558(0.318-0.979)	0.042	0.523(0.292-0.938)	0.03
Recessive Model						
AC+AA	192(100.0)	300(98.7)	1(ref)		1(ref)	
CC	0(0.0)	4(1.3)	NA	NA	NA	NA
Overdominant model						
AA+CC	173(90.1)	258(84.9)	1(ref)		1(ref)	
AC	19(9.9)	46(15.1)	0.616(0.349-1.087)	0.095	0.599(0.333-1.077)	0.087
rs1007888						
Codominant model						
CC	63(32.8)	85(28.0)	1(ref)		1(ref)	
CT	82(42.7)	149(49.0)	0.743(0.486-1.133)	0.168	0.740(0.475-1.152)	0.182
TT	47(24.5)	70(23.0)	0.906(0.553-1.483)	0.694	0.971(0.578-1.630)	0.911
Aelle model						
C	208(54.2)	319(52.5)	1(ref)		1(ref)	
T	176(45.8)	289(47.5)	0.934(0.723-1.207)	0.601	0.968(0.742-1.264)	0.812
Dominant Model						
CC	63(32.8)	85(28.0)	1(ref)		1(ref)	
CT+TT	129(67.2)	219(72.0)	0.795(0.537-1.176)	0.25	0.818(0.544-1.229)	0.333
Recessive Model						
CT+CC	145(75.5)	234(77.0)	1(ref)		1(ref)	
TT	47(24.5)	70(23.0)	1.084(0.709-1.655)	0.711	1.159(0.745-1.803)	0.514
Overdominant model						
CC+TT	110(57.3)	155(51.0)	1(ref)		1(ref)	
CT	82(42.7)	149(49.0)	0.775(0.539-1.116)	0.171	0.756(0.517-1.105)	0.149

By adjusting for pre-pregnancy body mass index, Systolic blood pressure, Diastolic blood pressure, age, and parity, logistic regression yielded the adjusted *p*-value.

### 3.5 The association between SNPs and GDM risk in individuals with a pre-BMI of 24 or above

In our investigation, we observed a compelling association between the *ARAP1* rs1552224 allele and a reduced risk of GDM specifically within a subset of females. Among those with a pre-BMI of 24 kg/m^2^ or greater, the presence of the C allele of the *ARAP1* rs1552224 SNP was significantly associated with 65.5% lower odds of developing GDM compared to carriers of the A allele (OR = 0.345, 95% CI: 0.124-0.960; *p* = 0.042). This finding, presented in **Table 5**, suggests a potential protective role of the *ARAP1* rs1552224 C allele in GDM susceptibility, particularly in the context of higher pre-BMI. Further research is warranted to elucidate the underlying biological mechanisms by which *ARAP1* may influence glucose homeostasis and GDM risk in this specific demographic.

**Table 5:** The correlations between SNPs and GDM risk in individuals with a pre-BMI of 24 or above.

Model	Cases (%) (n=192)	Controls (%) (n=304)	Crude OR (95% CI)	Crude *P*	Adjusted OR (95% CI)	Adjusted *P*
rs1552224						
Codominant model						
AA	89(91.8)	31(73.8)	1(ref)		1(ref)	
AC	8(8.2)	10(23.8)	0.279(0.101-0.769)	0.014	0.399(0.124-1.291)	0.125
CC	0(0.0)	1(2.4)	NA	NA	NA	NA
Aelle model						
A	186(95.9)	72(85.7)	1(ref)		1(ref)	
C	8(4.1)	12(14.3)	0.258(0.101-0.657)	0.005	0.345(0.124-0.960)	0.042
Dominant Model						
AA	89(91.8)	31(73.8)	1(ref)		1(ref)	
AC+CC	8(8.2)	11(26.2)	0.253(0.093-0.687)	0.007	0.337(0.108-1.048)	0.06
Recessive Model						
AC+AA	97(100.0)	41(97.6)	1(ref)		1(ref)	
CC	0(0.0)	1(2.4)	()		()	
Overdominant model						
AA+CC	89(91.8)	32(76.2)	1(ref)		1(ref)	
AC	8(8.2)	10(23.8)	0.288(0.104-0.793)	0.016	0.407(0.126-1.310)	0.132
rs1007888						
Codominant model						
CC	29(29.9)	11(26.2)	1(ref)		1(ref)	
CT	51(52.6)	23(54.8)	0.841(0.359-1.970)	0.69	0.841(0.336-2.104)	0.712
TT	17(17.5)	8(19.0)	0.806(0.271-2.397)	0.698	1.369(0.363-5.160)	0.643
Aelle model						
C	109(56.2)	45(53.6)	1(ref)		1(ref)	
T	85(43.8)	39(46.4)	0.900(0.538-1.505)	0.687	0.978(0.561-1.705)	0.938
Dominant Model						
CC	29(29.9)	11(26.2)	1(ref)		1(ref)	
CT+TT	68(86.1)	31(73.8)	0.832(0.369-1.877)	0.658	0.864(0.355-2.107)	0.749
Recessive Model						
CT+CC	80(82.5)	34(81.0)	1(ref)		1(ref)	
TT	17(17.5)	8(19.0)	0.903(0.356-2.292)	0.83	1.133(0.394-3.258)	0.816
Overdominant model						
CC+TT	46(47.4)	19(45.2)	1(ref)		1(ref)	
CT	51(52.6)	23(54.8)	0.916(0.443-1.894)	0.813	0.831(0.377-1.830)	0.646

By adjusting for pre-pregnancy body mass index, Systolic blood pressure, Diastolic blood pressure, age, and parity, logistic regression yielded the adjusted *p*-value.

Furthermore, our analysis did not reveal any significant associations between the genetic factors investigated and GDM across the other subcategories (**Supplementary Tables 1**–**3**). Furthermore, a one-way ANOVA was conducted to explore potential relationships between polymorphism genotypes and various blood glucose parameters. However, the associations between the genotypes of the SNPs and fasting plasma glucose (FPG), 1-hour post-glucose (1h-PG), and 2-hour post-glucose (2h-PG) levels were not statistically significant, as all *p*-values consistently exceeded 0.05 (**Supplementary Tables 4**–**6**).

### 3.6 Meta-analysis results

**Figure 1:**
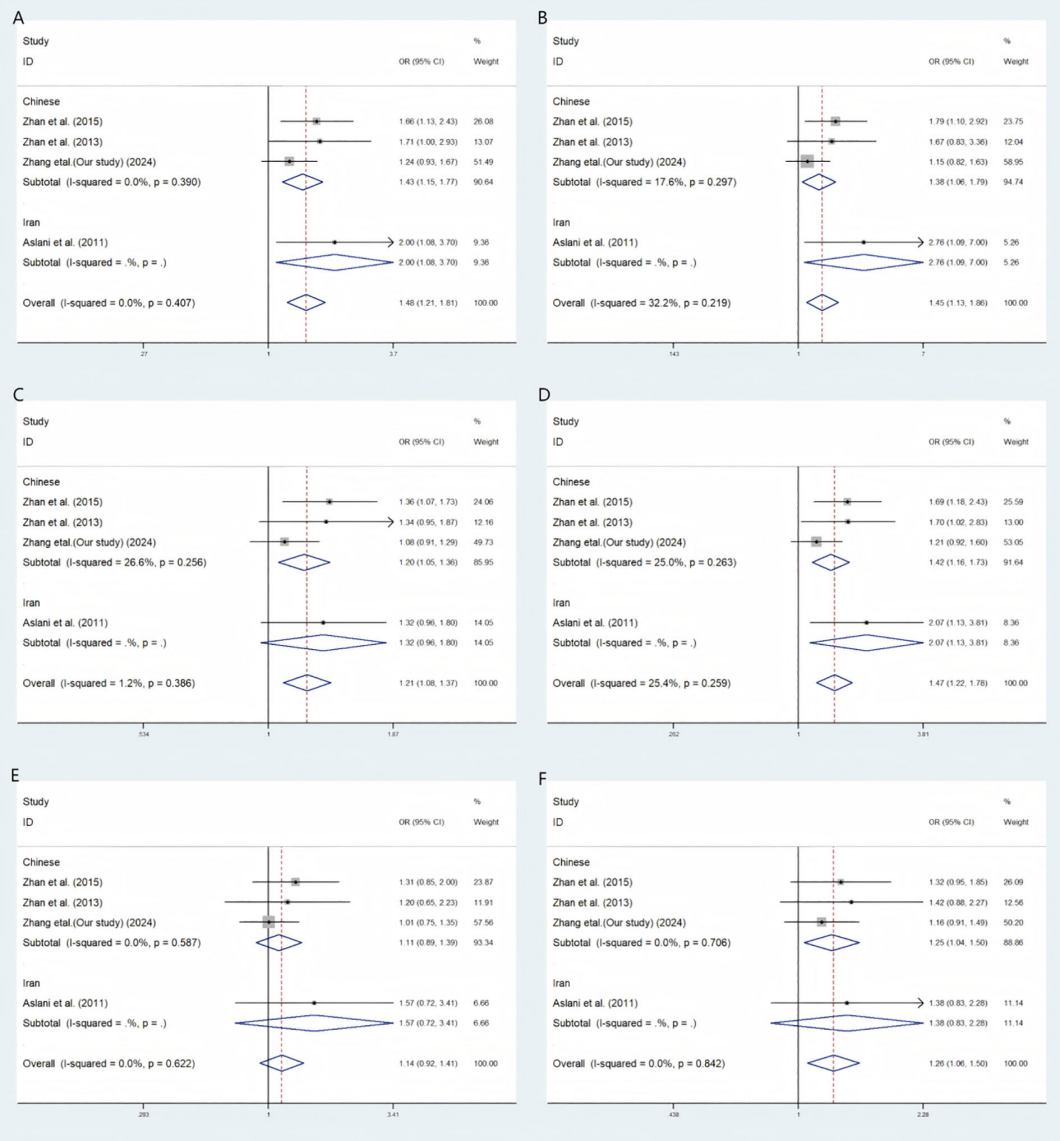
A meta-analytic method employing a fixed effects strategy to explore the link between *MIF* rs1007888 and the vulnerability to GDM. **(A)** Codominant Heterozygous Model, CC versus CT **(B)**. Codominant Homozygous Model, CC versus TT **(C)** Aelle model, C versus T **(D)** Dominant Model, CC in contrast to CT+TT **(E)** Recessive Model, CT+ CC versus TT **(F)** Over-dominant model, CC+TT versus CT. OR stands for odds ratio, CI for confidence interval, and I-squared represents the measure used to assess the level of diversity in the meta-analytic method.

**Figure 2:**
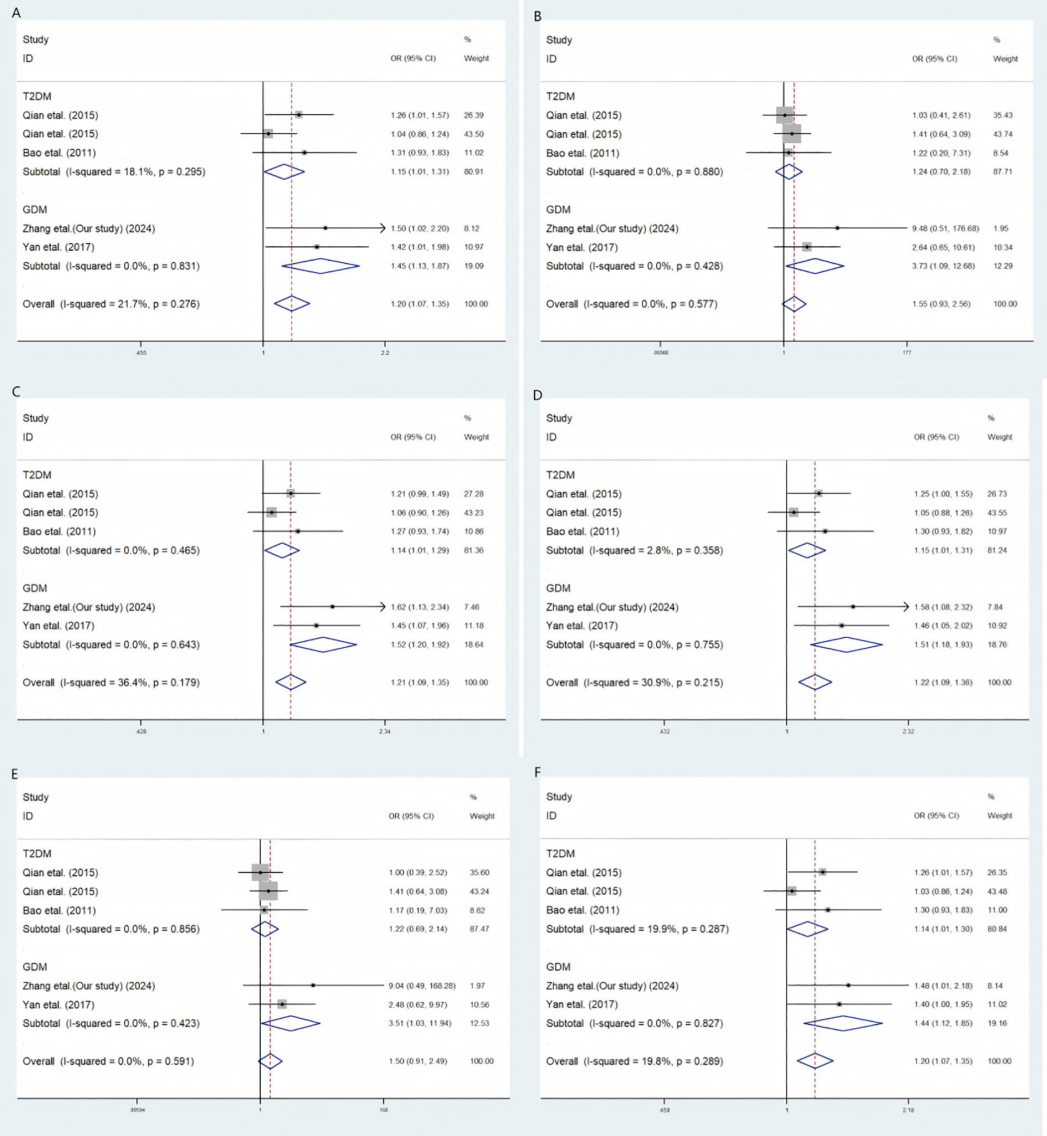
A meta-analytic method employing a fixed effects strategy to explore the link between *ARAP1* rs1552224 and the susceptibility to GDM. **(A)** Codominant Heterozygous Model, AA versus AC **(B)**. Codominant Homozygous Model, AA versus CC **(C)** Aelle model, A versus C **(D)**. Dominant Model, AA versus AC+CC **(E)** Recessive Model, AC+AA versus CC **(F)** Over-dominant model, AA+CC versus AC. OR stands for odds ratio, CI for confidence interval, and I-squared represents the measure used to assess the level of diversity in the meta-analytic method.

Our meta-analysis findings reveal a significant association between the rs1007888 polymorphism and an elevated risk of GDM across multiple genetic models, including the codominant heterozygote, codominant homozygote, allelic, dominant, and over-dominant models (**Figure 1**). This consistent association strongly suggests a role for rs1007888 in GDM susceptibility. Furthermore, subgroup analysis highlighted the rs1552224 polymorphism as significantly linked to an increased GDM risk in the codominant heterozygote, allelic, dominant, and over-dominant models. While the codominant homozygote and recessive models for rs1552224 did not show a broad overall effect on T2DM, they did exert a statistically significant influence specifically on GDM, with respective *p*-values of 0.035 and 0.044. Other genetic models for this polymorphism did not yield substantial correlations (**Figure 2**).

Regarding potential publication bias, Begg’s test indicated no substantial bias across the genetic frameworks (all *p* > 0.05). However, the observed asymmetry in the funnel plot (**Supplementary Figures 1**, **2**) warrants consideration. While this asymmetry could suggest publication bias, it’s also important to acknowledge that it may arise from other factors, such as underlying heterogeneity in study outcomes or variations in study size.

## 4 Discussion

Baseline characteristics from our cohort showed that women who developed gestational diabetes mellitus (GDM) had significantly higher systolic and diastolic blood pressure, fasting plasma glucose, 1-hour and 2-hour postprandial glucose levels, pre-pregnancy BMI, and mean age compared with controls (all p < 0.05, several p < 0.001). These observations are consistent with previous reports and point to several mechanistic underpinnings (18, 19).

Although none of the GDM participants met the diagnostic threshold for hypertension, their higher blood pressure values suggest early manifestations of insulin resistance–related endothelial dysfunction and subclinical inflammation (20). This pathophysiological background helps to explain the increased risk of preeclampsia and gestational hypertension in GDM pregnancies (4, 21).

The most pronounced group differences were seen in postprandial glucose excursions particularly at 1 and 2 hours supporting the concept that postprandial hyperglycaemia represents the core metabolic abnormality of GDM (4). This likely reflects inadequate first-phase insulin release and reduced peripheral glucose uptake.

Pre-pregnancy BMI emerged as another critical determinant. Even within the “normal” range (18.5–24 kg/m²), women who later developed GDM had higher pre-BMI values, suggesting that mild elevations in adiposity may confer risk. Adipokines such as leptin and TNF-α, secreted from adipose tissue, may further aggravate insulin resistance and impair β-cell function (22, 23).

Maternal age was also independently associated with GDM: the mean age of affected women was 31 years compared with 29 years in controls (p < 0.001), and stratified analysis showed that those aged ≥30 years had more pronounced metabolic impairments. This is consistent with evidence that both insulin sensitivity and β-cell function decline progressively with advancing age (4, 24).

Collectively, these findings underscore that even modest elevations in blood pressure, adiposity, and maternal age contribute to GDM risk. They highlight the need for pre-pregnancy counselling and weight optimisation in women of reproductive age as a preventive strategy to reduce the burden of GDM and its complications.

The human macrophage migration inhibitory factor (*MIF*) gene, located on chromosome 22q11.2, is constitutively and abundantly expressed in immune cells, particularly T lymphocytes and macrophages (25). *MIF* exerts diverse biological functions, and altered expression has been reported in several inflammatory and metabolic disorders, including rheumatoid arthritis, atherosclerosis, and diabetes (26). In the placental decidua, macrophages and dendritic cells regulate T-cell activation, initiating immune responses. Subsequent differentiation of T cells into Th1 or Th2 subtypes—driven by both intrinsic and extrinsic factors alters cytokine synthesis and distribution. These shifts directly influence *MIF* regulation and are essential for immunological adaptation across pregnancy stages (15).

Beyond its role in immunity, *MIF* functions as a pleiotropic cytokine linking inflammation to glucose metabolism. At the molecular level, it regulates glucose transporter 4 and fructose-bisphosphatase 2 expression (8). Moreover, *MIF* promotes TNF-α production (27, 28), thereby influencing glycolysis and inflammatory signalling. In adipocytes, *MIF*-driven TNF-α secretion contributes to insulin resistance (8), while in endothelial cells, *MIF* disrupts insulin-mediated nitric oxide release, fostering endothelial insulin resistance (8).

These mechanisms align with the lipid overload hypothesis, which posits that ectopic lipid deposition in insulin-sensitive tissues such as skeletal muscle, pancreas, and liver underpins insulin resistance (29–31). Supporting this, both adipose tissue *MIF* mRNA and circulating *MIF* concentrations are elevated in obese women with type 2 diabetes (32). Elevated adipose-derived *MIF* in obesity and diabetes further impairs insulin signalling and amplifies pro-inflammatory cascades, thereby reinforcing insulin resistance (33).

Despite previous findings linking *MIF* variants to T2D, our study found no significant association between the *MIF* rs1007888 variant and GDM. This divergence suggests that pregnancy-specific genetic or physiological factors might influence the role of this genetic locus in GDM development (6, 32, 33). Moreover, despite the known involvement of *MIF* in inflammatory and metabolic disorders (34), its genetic influence via rs1007888 on GDM risk within the context of higher pre-BMI appears to be negligible or absent in our specific cohort. This suggests that the genetic impact of rs1007888 on GDM risk may be either minor or does not significantly interact with the metabolic environment of pre-existing obesity, at least in the population we investigated. It is plausible that other genetic or environmental factors exert a more predominant influence on GDM development within this particular BMI subgroup.

*ARAP1*, which encodes the protein Arap1 (ARF-GAP, Rho-GAP Anchoring Protein Repeat Sequence, and Pleckstrin Homology Domain Protein 1, also known as centaurin delta 2), functions as a phosphatidylinositol 1,4,5-trisphosphate-regulated Arf GTPase-activating protein. This protein primarily targets the small GTP-binding protein Arf6, which is known to play a critical role in the modulation of insulin exocytosis (6). The rs1552224 SNP is situated within an intronic region of the *ARAP1* gene.

Research indicates that *ARAP1* is significantly associated with elevated blood sugar levels and reduced insulin secretion in response to glucose stimulation, suggesting that impaired pancreatic β-cell function may be a mechanism through which this genetic locus contributes to the development of diabetes (35). Recent findings propose that the expression of the *ARAP1* locus is predominantly influenced by markedly increased levels of *STARD10* expression in the pancreas (36).

The genetic variant *STARD10* rs11603334 is functionally linked to the expression of *ARAP1* and is in complete linkage disequilibrium with rs1552224. The risk allele (C) of *STARD10* rs11603334 for type 2 diabetes is positioned near the *ARAP1* promoter. This specific placement interferes with the collaborative complex formation between the transcriptional regulators PAX6 and PAX4. This interference leads to an increased amplification of *ARAP1* P1 promoter transcription, resulting in higher *ARAP1* transcription levels within the Islets of Langerhans (37).

Conversely, research by Carrat et al (36) suggests that compromised insulin secretion from pancreatic and β-cells is associated with decreased *STARD10* levels, rather than elevated *ARAP1*. Furthermore, the rs1552224C allele might offer a protective effect against type 2 diabetes. This protective role could be mediated by the overexpression of *STARD10*, which in turn enhances Ca^2+^ dynamics and glucose-triggered insulin release from pancreatic and β-cells (34). Despite these findings, the precise operational mechanism of this process in GDM requires further extensive investigation.

The protective influence of the C allele at rs1552224 warrants particular attention. *ARAP1* (ArfGAP with RhoGAP domain, ankyrin repeat, and PH domain 1) plays a critical role in insulin secretion and glucose homeostasis by regulating membrane trafficking and cytoskeletal dynamics within pancreatic β cells (38). While previous research has linked *ARAP1* polymorphisms to an elevated risk of type 2 diabetes (39, 40), its specific contribution to GDM, especially in younger populations, remains underexplored. Our findings suggest that the C allele may modify *ARAP1*’s function, potentially enhancing β-cell compensation or insulin sensitivity to meet the increased metabolic demands of pregnancy, thereby lowering GDM risk. This aligns with existing evidence that genetic variations affecting β-cell function are significant predictors of GDM susceptibility (41).

In the context of the allelic model for the *ARAP1* gene, the presence of each C allele is associated with a 38% reduction in the risk of developing GDM. This observation is consistent with the established role of *ARAP1* in insulin granule trafficking and the functionality of βcells (37). Notably, the absence of CC homozygotes among GDM cases (0% compared to 0.8% in controls) indicates a potential dosage-dependent protective effect. However, the low minor allele frequency (MAF = 5–8%) (42) may limit the statistical power of analyses based on recessive models.

Furthermore, our results corroborate previous functional studies that demonstrate that *ARAP1* knockdown adversely affects glucose-stimulated insulin secretion (GSIS) in β cells Benjamin (31). The protective C allele may enhance the expression of *ARAP1*, thereby improving the first-phase insulin response, which is crucial for regulating postprandial glucose levels. This is consistent with the elevated 1-hour and 2-hour post-glucose challenge levels presented in **Table 1**.

The observation that the rs1552224 C allele exhibits a protective association in individuals with elevated pre-BMI is a compelling finding. Given that obesity is a well-established driver of insulin resistance and a significant risk factor for GDM (43), our data imply that the C allele may offer a unique advantage by ameliorating the adverse metabolic consequences of obesity during gestation (38).

The SNP rs1552224 has the potential to enhance the prediction of GDM risk in women with high BMI and nulliparous status. However, its modest effect size, with an odds ratio of approximately 0.6, limits its utility as a standalone predictive marker. We hypothesize that this allele could modulate *ARAP1* function in a manner that either bolsters β-cell compensatory mechanisms or enhances insulin signaling pathways. This modulation would be particularly beneficial in counteracting the heightened insulin resistance characteristic of a higher pre-BMI, especially within a population already predisposed to GDM due to their initial weight. This interpretation aligns with the broader concept that genetic variants can influence an individual’s susceptibility to obesity-induced metabolic dysfunction (44).

A comparison of findings in **Table 3** with the data in **Table 5**, which utilized age as a stratification factor, indicates that the protective effect of the rs1552224 C allele is consistently observed across various risk stratifications, including age and BMI. This consistency enhances the potential clinical significance of this genetic variant as a biomarker for assessing the risk of GDM. Our research highlights the critical interaction between genetic factors and environmental influences, such as pre-BMI, in shaping susceptibility to GDM. By identifying genetic variants that may either increase or decrease the risk associated with obesity, we can develop more tailored strategies for the prevention and management of GDM. Future research should investigate the functional implications of rs1552224 and its potential role in targeted interventions for pregnant women with elevated BMI.

This meta-analysis provides a comprehensive assessment of the association between the rs1007888 variant and gestational diabetes mellitus (GDM), synthesising evidence from three independent studies (5, 45) together with data from Zhang et al. (our current study, 2024). The pooled analyses, illustrated in the accompanying forest plots (A–F), consistently demonstrate an increased risk of GDM associated with this locus, thereby offering new insights into the genetic basis of disease susceptibility.

In particular, **Figure 1** highlights that Plots A and B yielded statistically significant associations, with pooled odds ratios of 1.48 and 1.45, respectively. Importantly, both models displayed very low between-study heterogeneity (I² = 0.0% for A and 17.6% for B), underscoring the stability and reproducibility of these findings. Such homogeneity suggests that the genetic effect captured in these models—likely reflecting an additive or dominant contribution of the risk allele—represents a robust and biologically meaningful determinant of GDM.

The consistency of associations across both Chinese cohorts (5, 45) and an Iranian cohort (6) further strengthens the evidence, supporting the notion of a shared genetic predisposition that transcends ethnic boundaries. This aligns with prior meta-analyses reporting that common genetic variants contribute significantly to GDM risk across diverse populations (26, 41). The low heterogeneity observed in several key models adds to the reliability of our effect estimates and suggests that rs1007888 may represent a stable genetic marker of GDM susceptibility.

Our study has several significant limitations that should be acknowledged. First, the relatively small number of studies included in the meta-analysis restricts the strength of the conclusions. This underscores the need for larger, multi-ethnic investigations to validate our findings and to explore potential gene-environment interactions. Functional studies will also be essential to elucidate the biological mechanisms by which these variants may contribute to GDM risk. Ultimately, identifying reliable genetic markers could inform personalized risk stratification, early screening, and targeted prevention strategies for GDM.

Discrepancies were observed between the results of our case–control analysis, the meta-analysis, and previous literature. We propose that a limited sample size represents a key contributor to these inconsistencies. Power calculations using G*Power software indicated that approximately 19,788 participants would be required to assess the association with rs1007888 adequately, and 2,273 participants for rs1552224, under the following parameters: test family = Exact; statistical test = Proportions: Inequality, two independent groups (Fisher’s exact test); test direction = two-tailed; power = 0.8; significance level α = 0.05.

The challenges of sample size requirements differ between variants. For rs1552224, the low minor allele frequency (0.079) limits statistical power, necessitating large cohorts to detect associations. For rs1007888, although the reference allele is more common (p2 = 0.486), the effect size is minimal (OR = 0.923), which again demands a substantial cohort to achieve reliable power. This highlights a broader difficulty in genetic association studies of weak-effect variants, where enormous sample sizes are often required to detect modest associations.

In addition to sample size, the absence of comprehensive adjustment for potential confounders may have contributed to the discrepancies observed. Significant clinical and biochemical variables such as lipid profiles, fasting glucose, HOMA indices, C-peptide concentrations, and comorbidities were not systematically included. Moreover, the lack of multicenter data collection limits generalizability, as regional differences within China may influence allele frequencies and disease risk. Addressing these issues in future studies through larger sample sizes, standardized adjustment factors, and multicenter recruitment will be crucial to resolving these inconsistencies and advancing understanding of the genetic basis of GDM.

## 5 Conclusions

This study investigated the relationship between the *MIF* rs1007888 and *ARAP1* rs1552224 genetic variants and the risk of GDM. Our findings revealed no significant association between the *MIF* rs1007888 variant and GDM risk. *ARAP1* rs1552224 was significantly linked to reduced GDM incidence. In contrast, meta-analysis results indicated that the *MIF* rs1007888 mutation was associated with an increased likelihood of developing GDM. Furthermore, the *ARAP1* rs1552224 mutation was linked to a heightened risk of GDM. The discrepancies between our findings and those of previous studies may be attributed to limitations in sample size and ethnic diversity. To enhance the understanding of the relationship between these genetic variants and GDM risk, future research should prioritize increasing sample sizes and incorporating a broader range of adjustment factors and diverse ethnicities.

## Data Availability

The datasets presented in this study can be found in online repositories. The names of the repository/repositories and accession number(s) can be found in the article/**Supplementary Material.**
