# Supplementary material for: Association of MIF rs1007888 and ARAP1 rs1552224 genetic variants with the risk of gestational diabetes mellitus in a chinese population; case study and meta-analysis
Source: Front Endocrinol (Lausanne). 2025 Sep 16;16:1650782. doi: 10.3389/fendo.2025.1650782 (PMC12479318; doi:10.3389/fendo.2025.1650782)
Supplement: Supplementary file 1 [file DataSheet1.docx]

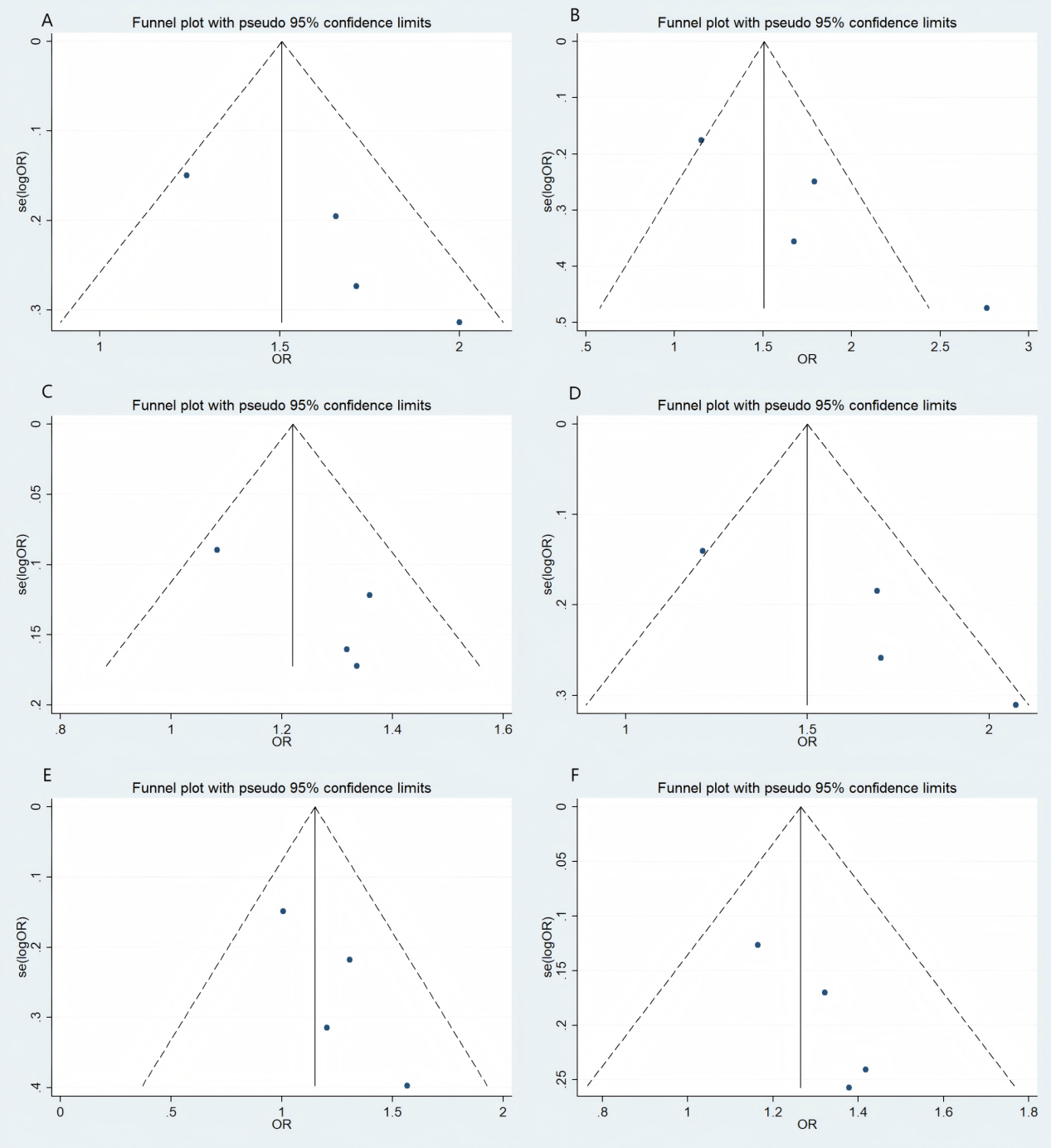


Supplementary Figure 1

Funnel diagrams serve to identify possible biases in publication. (A) prevailing model, CC versus TT+CT (B) recessive framework, CT+CC versus TT (C) predominant model, TT +CC versus CT (D) represents a codominant homozygous model, CC in contrast to TT (E) a codominant heterozygous model, CC versus The CT (F) allele framework, C versus (T) OR stands for odds ratio, and CI represents confidence interval.


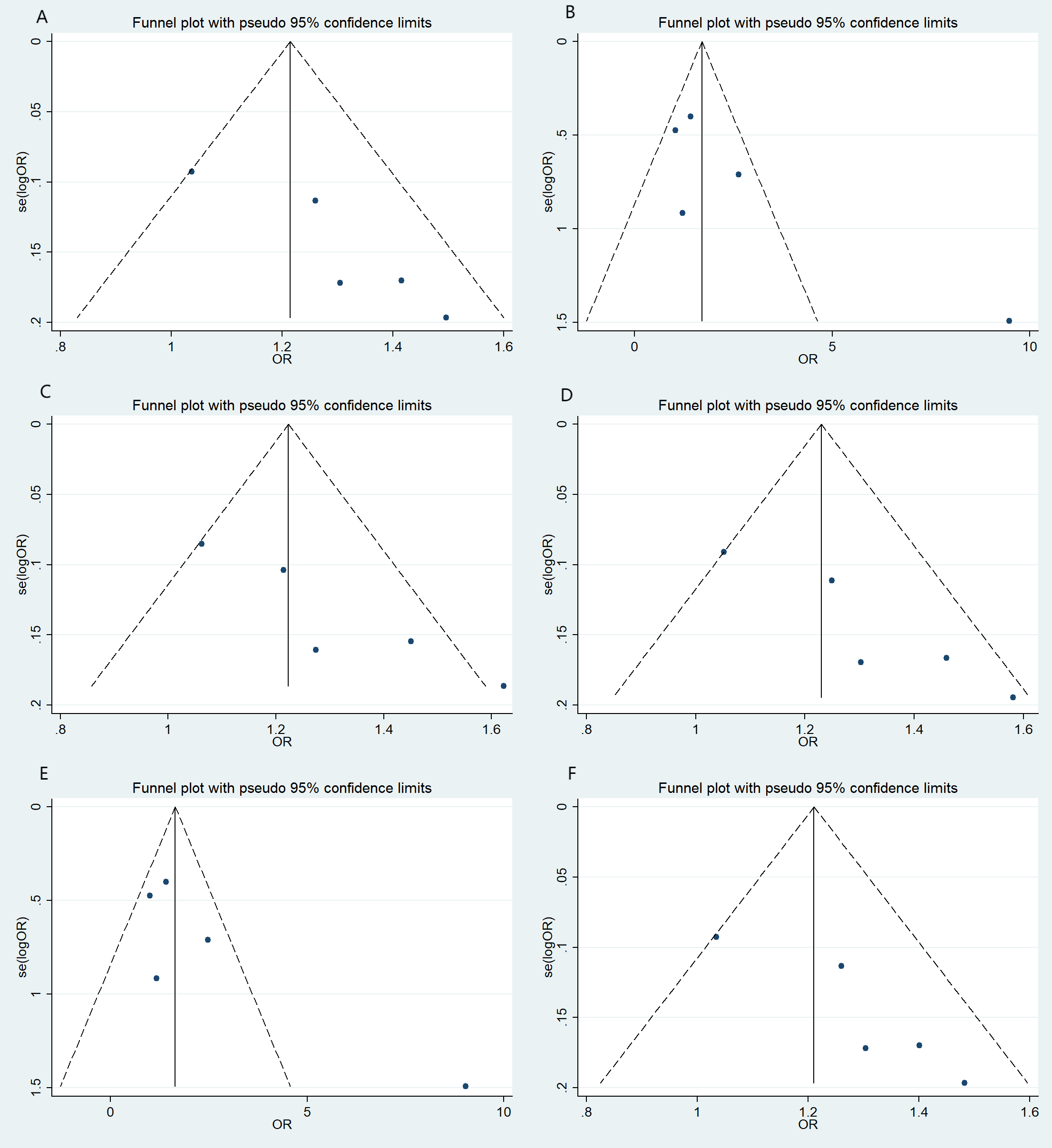


Supplementary Figure 2

Funnel plots serve the purpose of identifying possible biases in publications. (A) prevailing model, AA versus AC+C (B) recessive model with AC+AA. CC (C) predominant model, AA +CC versus AC (D) represents the co-dominant homozygous model, AA versus CC (E) denotes the codominant heterozygous model, AA versus AC (F) allele model contrasts A with. (C) Odds Ratio (OR), Confidence Interval (CI).
